# Sustaining Knowledge Translation Practices: A Critical Interpretive Synthesis

**DOI:** 10.34172/ijhpm.2022.6424

**Published:** 2022-02-21

**Authors:** Robert A.J. Borst, Rik Wehrens, Roland Bal

**Affiliations:** Erasmus School of Health Policy & Management, Erasmus University Rotterdam, Rotterdam, The Netherlands.

**Keywords:** Knowledge Translation, Institutionalisation, Sustainability, Context, Critical Interpretive Synthesis, Review

## Abstract

**Background:** The health policy and systems research literature increasingly observes that knowledge translation (KT) practices are difficult to sustain. An important issue is that it remains unclear what sustainability of KT practices means and how it can be improved. The aim of this study was thus to identify and explain those processes, activities, and efforts in the literature that facilitate the sustaining of KT practices in health policy-making processes.

**Methods:** We used a critical interpretive synthesis (CIS) to review the health policy and systems research and Science and Technology Studies (STS) literature. The STS literature was included as to enrich the review with constructivist social scientific perspectives on sustainability and KT. The CIS methodology allowed for creating new theory by critically combining both literatures. We searched the literature by using PubMed, Google Scholar, Web of Science, and qualitative sampling. Searches were guided by pre-set eligibility criteria and all entries were iteratively analysed using thematic synthesis.

**Results:** Eighty documents were included. Our synthesis suggests a shift from sustainability as an end-goal towards sustaining as actors’ relatively mundane work aimed at making and keeping KT practices productive. This ‘sustaining work’ is an interplay of three processes: (*i*) translating, (*ii*) contexting, and (*iii*) institutionalising. Translating refers to activities aimed at constructing and extending networks. Contexting emphasises the activities needed to create contexts that support KT practices. Institutionalising addresses how actors create, maintain, and disrupt institutions with the aim of sustaining KT practices.

**Conclusion:** The ‘sustaining work’ perspective of our CIS emphasises KT actors’ ongoing work directed at sustaining KT practices. We suggest that this perspective can guide empirical study of sustaining work and that these empirical insights, combined with this CIS, can inform training programmes for KT actors, and thereby improve the sustainability of KT practices.

## Background

 The past decades have shown a surge of studies and practices that seek to improve the use of health research in policy and practice. Within the health policy and systems research literature, this field is commonly referred to as ‘knowledge translation’ (KT). KT scholars and practitioners underscore the importance of both evidence-informed policy-making and practice, and policy- and practice-informed evidence generation.^1,2^ The KT field has gained substantial knowledge of the workings of KT practices – such as policy dialogues and the creation of rapid review services.^3,4^ Yet, such practices and their outcomes prove notoriously difficult to sustain: review services may be halted and policy dialogues may result in a temporary intention to change policy only.^5-7^ This lack of sustainability is often ascribed to the temporary and tentative nature of the research or implementation projects as part of which the KT practices were initiated.^8^ The health policy and systems research literature emphasises that the sustainability of KT practices may be even more at risk in low- and middle-income countries, where KT work is often conducted as part of donor-funded programmes that might not take the local knowledge and policy contexts into account.^9,10^

 Despite the increasing emphasis on sustainability of KT practices, there remains significant conceptual unclarity in the health policy and systems research literature over what sustainability means. Such conceptual unclarity impairs our understanding of why some KT practices do sustain, or how their sustainability can be improved. One of the conceptual approaches in the health policy and systems research literature sees sustainability of KT practices as the extent to which they are routinised, or exist over time.^5,6,11^ This means, for instance, that policy-making processes would be regularly informed by relevant knowledge through interactions between policy-makers, researchers, representatives from civil society organisations, and other involved actors.^12^ Others suggest that sustainability of KT practices depends on how they are organised, or structured. This part of the health policy and systems research literature conceptualises KT platforms as a sustainable way of organising KT practices.^13,14^ KT platforms are organisational forms that provide home to the actors that do KT work and function as a place where policy, research, and practice actors can interact.^4,15^ There is little agreement on how these different approaches relate, and more importantly: what kind of work is necessary to achieve and maintain these types of sustainability.

 Health policy and systems research that does focus on how sustainability of KT practices can be achieved, commonly identifies ‘factors’ for sustainability. Most prominent in such studies are institutional or contextual factors. Institutional factors often address the importance of efficient governance, local embedding of KT practices, and the presence of legislation in favour of evidence-informed policy-making.^12,16^ Analyses into the function of context for sustainability produce yet a different set of factors, such as: stable funding for KT work, adequate KT facilities, and a recipient environment in favour of evidence-informed practice.^12,17^ The lists of factors usually differ across settings, with some authors concluding that this means that they should be seen as mere guidance and not as prescriptive factors.^18,19^ While valuable in terms of reducing complexity, a key problem with the factor-approach is that it offers little information about the kind of work that is required to construct such factors in the first place.^20,21^ Instead, the factor-approaches provide snapshots of what sustainability under specific circumstances and at specific times and places may look like. Understanding how KT practices are *made* sustainable, and what that sustainability involves, requires a conceptual shift towards a more dynamic and practice-centred perspective on sustainability.

 To create a more dynamic and practice-centred perspective on the sustainability of KT practices, this study seeks to synthesise health policy and systems research perspectives with insights from Science and Technology Studies (STS). These literatures work from different epistemological standpoints, with the former being largely realistic and positivist and the latter generally being more constructivist in nature. Yet both literatures revolve around questions of KT and sustainability. A synthesis of these literatures has the potential to create a more coherent theory on sustainability of KT practices and produce insights into the work that goes into sustaining KT practices. Thus, the aim of this study was to review the health policy and systems research and STS literatures on sustainability and KT and identify and explain those processes, activities, and efforts that facilitate the sustaining of KT practices. The insights from this study can inform future empirical studies into the sustainability of KT practices, and the organisation of skill-building programmes that explicitly take sustainability into account.

## Methods

###  Design

 Our literature review did not aim for a neutral aggregation of literature, but sought to produce new theoretical insights into the sustaining of KT practices by combining insights from diverse literatures. We specifically chose to review both the health policy and systems research literature and the STS literature. The former was selected because of its explicit familiarity with KT activities and methods, whereas the latter literature was chosen because of its constructivist appreciation of mundanity – specifically its focus on what actors *do in practice *to produce, utilise, and translate knowledge.^22,23^ We used the critical interpretive synthesis (CIS) approach to a literature review, because both literatures are heterogenous, span decades of work published both in books, scientific articles, essays, and reports, and our aim was explicitly to interpret and combine their heterogeneous theoretical backgrounds into new insights.^12,14,24^

**Figure 1:**
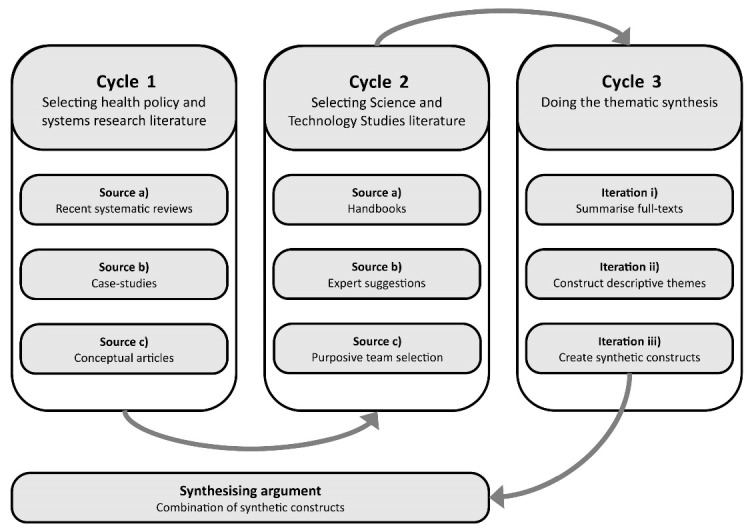
Overview of the Review Cycles.

 Following Dixon-Woods et al,^24^ our CIS consisted of three cycles (see Figure 1). First, we systematically searched relevant health literature databases and identified all (*a*) recent systematic reviews, (*b*) case-studies, and (*c*) conceptual articles that related to the sustainability of KT practices. The second cycle involved mapping all relevant literature in STS, whereby we focussed on what insights have been developed about durable interactions between research, policy, and practice. The literature searches involved a selection procedure guided by pre-set eligibility criteria (see Supplementary file 1). Third, we analysed the records through thematic synthesis – which produced a set of key descriptive themes. Using these themes, we developed so-called synthetic constructs. Synthetic constructs bind together the different themes and provide a new conceptual interpretation of the existing materials.^24^ In turn, these synthetic constructs together composed the synthesising argument. This synthesising argument is the key output of the CIS and offers a narrative that explains the connections between the synthetic constructs and a holistic interpretation of the reviewed materials.

###  Compass Question

 We formulated a compass question that would guide the design and conduct of the synthesis.^24,25^ The compass question we used was: which insights from the STS literature can help in better understanding how KT practices in the health policy- and health systems sector can be sustained? As such, our focus was on the international domain, rather than on individual organisational levels.

###  Literature Searches

 The literature searches were conducted between December 2018 and February 2019, followed by an update in December 2020. In the first cycle, we used three strategies to identify relevant records within the health policy and systems research literature. First, we operationalised the compass question into systematic search phrases to screen PubMed for systematic reviews on KT practices and their sustainability (search phrases are provided in Supplementary file 1). Second, we used search phrases to identify case-studies via PubMed that specifically focussed on sustainability in relation to KT practices. Finally, we conducted additional searches for conceptual KT sources that were not included in PubMed, by using Web of Science and Google Scholar.

 The second cycle of the review specifically concentrated on the STS literature. As the field of STS spans several decades of work, we limited our search to: (*a*) a review of the three handbooks on STS, (*b*) a selection of core texts suggested via interviews with independent experts, and (*c*) a purposive selection of texts as identified through deliberation among the review team members. Handbooks often serve to summarise key ideas and concepts in a discipline,^26^ and the widely used STS handbooks include chapters by contemporary key-scholars. As such, the historical overview of the field in these handbooks allowed us to identify key chapters that address relevant theoretical background on issues of sustainability of KT practices. All authors were involved in reviewing chapters from the three handbooks. Using predetermined inclusion and exclusion criteria, we independently provided recommendations to include or exclude a chapter. During a subsequent two-hour reflectional meeting, deviations in our selections were discussed and deliberated on. All chapters with at least two recommendations for inclusion were selected for the review.

 By relying on handbooks, our selection may be skewed to European and Northern-American scholars only – something STS has been criticised for.^27^ We therefore asked ten experts in STS from different genders and continents to propose up to three texts each that they consider to be crucially important for understanding how to sustain KT practices. We repeated the procedure among the three researchers involved in the synthesis and discussed the outcome of this procedure in a three-hour consensus meeting.

 A complete overview of the search strategy and all syntaxes used in selecting the literature is available in Supplementary file 1.

###  Inclusion and Exclusion

 After the first selection of potentially relevant literature, all sources were screened by the research team. For the health policy and systems research literature, this involved a reading of title and abstracts by RAJB. Using pre-set inclusion and exclusion criteria (see Supplementary file 1), we then removed irrelevant and duplicate papers from the selection. We used a different screening approach for the STS literature – as these sources were mostly books, book chapters, or scientific articles without abstract. Thus, we divided these records for screening over the researchers involved in the review. The reviewers then independently wrote short summaries of these records that explained the problem statement and key concepts. These summaries were used to deliberate on the relevance of the records during two meetings.

###  Data Analysis and Synthesis

 The final cycle of our CIS involved thematic synthesis and the construction of a synthesising argument respectively. The analysis of selected records evolved through three iterative stages that we based on Dixon-Woods et al,^24^ Schutz,^28^ and Thomas and Harden.^29^ First, we read all full texts and further summarised the main argument and key concepts. We used these summaries to construct and connect descriptive themes that stayed very close to the original texts. The final step was to create the synthetic constructs – which we did by constant comparison between the original texts and the descriptive themes. We discussed in the team how the themes might connect and what combination allowed for a holistic interpretation of most data. The outcomes of the analyses were constantly cross-checked with members from the research team that were not directly involved in this review, but did have extensive experience studying and organising KT practices. The core constructs were developed by combining the descriptive themes. We subsequently produced the synthesising argument by relating the synthetic constructs into a coherent conceptual framework. Following common practice in qualitative research, we kept an audit trail and used this to prepare the manuscript.^30^

## Results

 The findings of the review are divided into three parts. First, we discuss the search results and the selection of records. Second, we summarise the overall contribution of our framework (ie, the synthesising argument, cf. Dixon-Woods et al^24^) and specify how it allows for a better understanding of the sustainability of KT practices. Last, we describe in detail how the different concepts from STS and the health policy and systems research literature contribute to our conceptual framework and what role they play in the sustainability of KT practices.

###  Search Results

**Figure 2:**
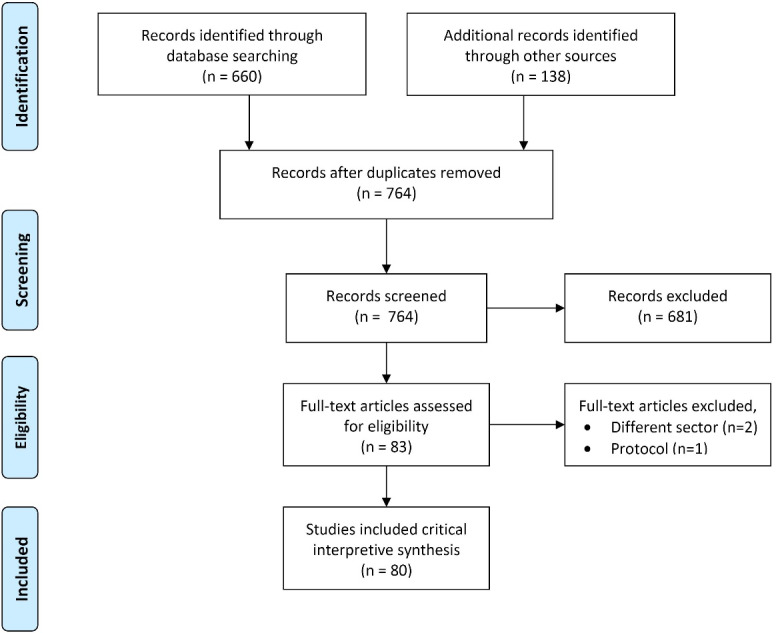
PRISMA Chart Showing How the Selection of Records Was Arrived at.

 Our bibliographic search comprised six different sources. After deduplication, the six sources left us with a first selection of 764 records. These records were reviewed for their relevance by applying our inclusion and exclusion criteria (see Supplementary file 1). This excluded 681 records for various reasons, most notably because they focussed on clinical practice only, did not address KT, or the full texts were unavailable. In total, 80 records, of which 38 from the STS literature, were included in the final analysis (see Figure 2).

###  Synthesising Argument

 In our problem statement we addressed that the health policy and systems literature on KT has produced detailed lists of factors that should be considered when organising sustainable KT practices. Such lists often mention that context is important and that institutional arrangements should be considered. At the same time, these lists offer little description of how sustainability is achieved *in practice*. The synthesising argument of this CIS is that conceptualisations of sustainability of KT practices would benefit from a shift from viewing sustainability as an end-state, produced through a list of factors, towards *sustaining as the (often mundane) work that is required to make and keep KT practices productive*. This means that sustainability as such should not be viewed as a state, but rather as a set of *ongoing activities. *Sustaining thus becomes a process without a clear end – one that is moreover an inherent part of KT practices. To emphasise the practical efforts that are necessary to sustain KT practices, we describe this set of activities with the term *sustaining work*. Based on the literature, we distinguish between three processes of sustaining work: (*i*) translating, (*ii*) contexting, and (*iii*) institutionalising KT practices. In the subsequent sections, we will explicate for the three processes how the perspective created through this CIS differs from more conventional health policy and systems research perspectives on sustainability.

###  Translating

 Since the early 21st century, the health policy and systems research literature has seen a rapid increase in the use of the term ‘knowledge translation.’^31^ Initially, Lomas^32^ spoke of translation with an emphasis on communication – especially making research knowledge more understandable to policy-makers. Over time, KT gradually became depicted as an iterative and dynamic process that aimed to increase the use of research knowledge in policy and practice.^33^ However, the health policy and systems literature often leaves the word ‘translation’ unproblematised and uses the term as synonym for ‘transfer,’ ‘exchange,’ and ‘mobilisation.’^31,34^ Without specifically tracing the entire etymology of ‘translation,’ we will show how the STS understanding of translation provides deeper insight into the sustaining of KT practices by highlighting that KT refers to a combination of transforming knowledge *and* creating new connections between actors that produce and utilise knowledge.

####  Different Understanding of Translation

 Translation as described in STS has a different meaning than in most of the health policy and systems research literature. This is partly due to the specific use and meaning of the word in French and Latin.^34^ In French, *translation* connotes both transformation and displacement.^35^ Within STS, this emphasis on transformation and displacement is used to describe how networks of actors are made, and often changed, in the process of knowledge production and utilisation. Callon^36^ described in a seminal paper on translation how this process can be characterised in four moments, that is: problematisation, interessement, enrolment, and mobilisation. These moments describe how actors first gather around a problem and potential way forward. Interessement then can be conceived as the moment that “influential actors” are linked to this potential way forward.^36^ Subsequently, these actors need to be assigned a role that describes what their responsibilities are. The final moment is where these actors are mobilised and made to play their role. This understanding of translation is part of actor-network theory (ANT) and underscores the constant (re-)building of networks and (strategic) work of transformation and displacement. Notably, what is an ‘actor’ in this regard is not stable nor confined to either social or material entities. To become an actor – that is, a human or non-human entity that can influence a course of events – needs work in itself.^36^

 While initially used to study power relations and the development of technologies,^36-39^ the STS understanding of translation was later widely applied to studies of knowledge production and utilisation. In particular, it has been used to better comprehend why scientific knowledge is not easily and directly applicable to places and situations other than those where the knowledge was produced. Following Latour^35^ and Callon,^40^ the production and utilisation of knowledge generally can be described in three translations. These three translations are each composed of the different moments as described above. The first translation happens when researchers attempt to bring something from the world into somewhat secluded and protected research spaces – think of blood samples or population data. Having retrieved their study materials, researchers work on a second translation where they manipulate properties of the study subject and expose it to all kinds of tests. The research space is made to resemble the outside world as much as reasonably possible, but is at the same time meant to protect against distortion from the outside world. This is comparable to how health scientists conduct randomised controlled trials on new interventions: test subjects are often asked to abide by a strict research protocol or regimen while still partaking in regular life. In the third translation, the researchers may aim to ‘implement’ their knowledge into existing practices. But knowledge does not unproblematically move from the secluded research space to the outside world: existing practices need to change and the conditions under which the knowledge was produced in the research space need to be reproduced in the utilisation environment as well.^41^

####  Translation and the Transfer of Knowledge

 The STS literature has conceptualised the process of translation in different ways. The key difference between how translation is used in STS and its use in most of the health policy and systems research literature, is the emphasis on the (re)construction of so-called actor-networks. In ANT, the world is deemed to be composed of humans *and* things who can ‘act’ (together referred to as: actors or *actants*). These actors are bound together in networks, and such networks are constantly (re)created through translations. Earlier works have deconstructed the notion of translation into separate moments or phases.^35,36^ What contemporary STS contributions have in common is a focus on the *places* of translation, for instance in the production and utilisation of knowledge.^42,43^ It is this understanding in particular that might inform efforts directed at sustaining KT practices in health policy-making processes.

 In Callon et al,^43^ the ‘sociology of translation’ is revisited. The authors describe that most types of research are no longer as secluded as they used to be. Apart from research in protected ‘laboratories’ (eg, randomised controlled trials), it has become more common to conduct ‘research in the wild.’ The ‘wild’ is meant to connote co-productive practices where knowledge is produced through interactions between secluded research and more open forms of research. Examples include citizen science and types of participatory action research. In her work on co-production, Jasanoff^44^ goes as far as to state that scientific knowledge is *always* co-produced, as interaction between researchers and other actors is inherent to doing research – albeit sometimes less explicitly so.^45^ Following the logic of co-production, translation is also about connecting and extending the (actor-) networks between knowledge production and -utilisation.

####  Translating and the Sustaining of Knowledge Translation Practices

 The STS understanding of translation brings two crucial insights to the health policy and systems research literature. The STS literature on translation suggests that KT is both about transforming knowledge as to make it utilisable, *and* about creating connections between places of knowledge production and places of knowledge utilisation that were not there before. This opposed to the health policy and systems research literatures that approaches KT as “a dynamic and iterative process that includes the synthesis, dissemination, exchange and ethically sound application of knowledge.”^33^ The STS emphasis on connections is important, because they carry the knowledge between the different actors, and thus their productivity seems a prerequisite for doing and sustaining the KT efforts. This act of translating is somewhat comparable to what earlier health policy and systems research scholars have identified as the ‘informal’ part of linkage and exchange approaches,^46,47^ where (strategic) partnerships are actively constructed and maintained through informal collaboration. Following the STS approach to translation, such approaches are not merely informal side-activities, but an essential part of ‘formal’ translation work.

 Second, the STS understanding of translation reiterates that (scientific) knowledge is not directly applicable for health practitioners and policy-makers – even when that knowledge is transformed in a for such communities appropriate and accessible way. The STS literature describes instead that knowledge, through its production, is always inscribed with assumptions about the environment in which it is to be used. Translating knowledge to health policy audiences then requires an opening of that black-boxed knowledge, and a mutually adaptive process where the knowledge is adapted to this new environment and the environment resembles the circumstances under which the knowledge was produced.^48^

 In short, the STS literature suggests that *translating *takes an important role in the sustaining of KT practices. This work involves creating networks between knowledge producing communities and actors that may be seen as intended users of such knowledge, and a mutually adaptive process where both the knowledge and its supposed utilisation environment are aligned with each other.

###  Contexting

 The health policy and systems research suggest that context plays an important role in sustaining KT practices. Within this literature, context is often seen as the conduciveness of a given environment to the implementation and routine conduct of certain KT practices. This means that context is a characteristic of that environment which is external to the KT practices themselves and that impacts those practices. The STS literature has traditionally approached context as a non-issue: context is a line in the sand that the ‘implementers’ of a KT practice constructed to define their intervention and the environment of the intervention. Recent STS scholarship, however, has opened the discussion on context again and these insights may help in better understanding the role of context in sustaining KT practices. We will start this section by briefly presenting how the health policy and systems research literature conceptualises context and will subsequently describe what insights from the STS literature may enrich this perspective.

 Our analysis shows that the health policy and systems research generally conceptualises the context of KT practices in two different ways. First, context is characterised as a local environment to which the KT practice needs to be attuned.^49,50^ This process is often referred to as contextualisation, which refers both to adding ‘local context’ to the KT practice itself as to make it more effective (eg, presenting research knowledge in a way that is common in that specific environment), and to changing the local context to be more conducive to the KT practice. Second, context is defined as a set of contextual attributes that may act as facilitator or barrier when implementing a KT practice or other intervention. Commonly identified factors, or attributes are for instance ‘financial context’ or ‘cultural context.’^12,51-53^ More generally, this perspective defines context as the “characteristics of the setting surrounding an organisation in which the implementation takes place.”^53^ In this second approach, context is clearly external to the KT practices, or in the words of Squires et al^17^: “(…) factors that are separate from the actual intervention itself and the actors receiving the intervention, but which may nonetheless contribute to the success of the intervention.”

####  Context as Network

**Figure 3:**
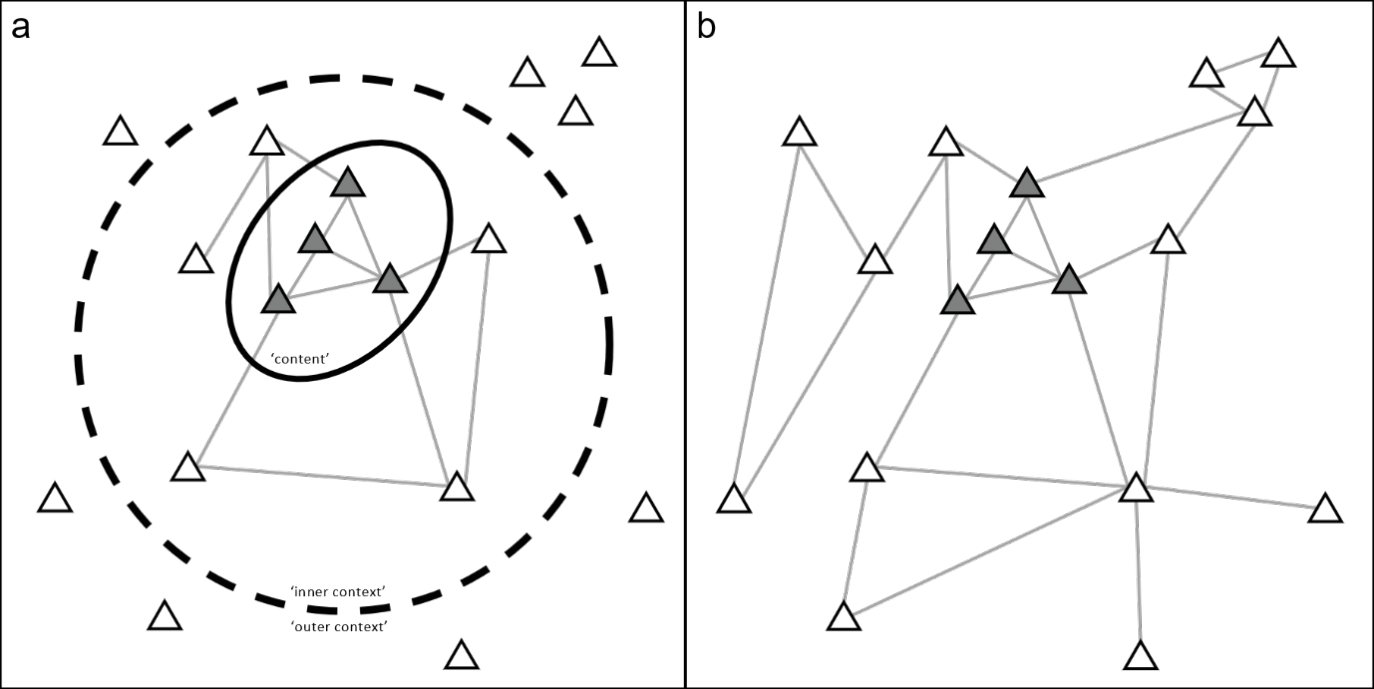
Understandings of Context. (a) shows a situation where content actors (grey triangles) are seen as conceptually distinct (solid circle) from contextual actors. The context itself is often divided in an inner context, where actors are related to the content, and an outer context that is seemingly disconnected from the content but nonetheless affects it. The alternative in (b) shows a flat ontology where all actors are connected, but an emphasis on certain parts of that network (grey triangles) can be applied – this does not require a conceptual delineation between content and context. Yet the actors outside this emphasis (white triangles) can, in their relationality, very much play a contextual role.

 The review of the STS literature proposes a conceptualisation where KT practices and context are inherently part of the same network. This conceptualisation builds on the ANT literature within STS.^54,55^ ANT works with three propositions, namely that: (*a*) the world exists of many intertwined networks, (*b*) these networks are constantly being (re)built, and (*c*) the nodes in the network are not merely humans, but also ‘things,’ or non-humans. These networks can be changed, with new actors being added or others being removed. This means that KT practices cannot be seen independently from the wider network in which they work (see Figure 3b). At the same time, the KT practices themselves can be seen as a web of different actors, such as policy-makers, policy briefs (ie, a synthesis of knowledge in a form appropriate for policy audiences), invitation letters, meeting venues, etc. As such, the KT practices are not easily distinguishable from their wider environment. Usually, however, the KT practices tend to be separated for analytical purposes, or when issues or ‘barriers’ – such as shortages in funding or insufficient organisational support – arise. In short, this STS conceptualisation of KT practices and context argues that it is not a matter of adding context to a KT practice (cf. contextualisation), but assigning some parts within the network of the KT practice a role as context.^56,57^

 In the STS understanding of ‘contexting,’ the emphasis is on the process in which actors are assigned a role as context in a given network.^57^ Our synthesis shows that this network conceptualisation to context still allows for speaking of ‘things in their context.’ But this context does not have a different status than the content it supposedly encompasses. Content and context are two different labels that refer to the same network of actors, yet the label ‘content’ singles out a specific group of actors within this network while excluding others. Thus, as has been argued before,^57,58^ what may be considered context and how context plays a role in KT practices is not something fixed. Instead, the role and boundaries of context are subject to continuous negotiation and judgment.^59^ This also means that what is seen as context in one instance, can be content in another. For example, when KT practitioners seek to inform the development of new policy on a health issue they commonly construct a boundary between evidence-based interventions (the content) and elements that could distort this content, such as funding, politics, and infrastructure (the context). The contexting perspective would argue that these ‘contextual elements’ are very much connected to the content, and may even describe its productivity (cf. Dixon-Woods et al^60^). It only becomes possible to distinguish content from context by tracing how the boundaries between the two are constructed.

####  Contexting and the Sustaining of Knowledge Translation Practices

 This CIS has presented a new perspective into the role of context in sustaining KT practices. The network approach to context that is used in the STS literature (ie, contexting) emphasises that KT actors must constantly *construct contexts that work* for their practices to remain productive. STS scholars Law and Moser^61^ use the notion of ‘patchworks’ to metaphorically describe that contexting is about knitting together actors in such a way that ‘the fabric’ (ie, the KT practice) becomes more sturdy. This is an important observation in relation to the sustaining of KT practices, as this suggests that the practices attain more stability through KT actors’ ability to make contexting an explicit part of their activities. In other words: it seems crucial for KT practices to enrol contextual actors in such a way that it helps them to sustain. In that perspective, the boundary between what is considered content and context is a political and strategic one, which is constantly re-negotiated and re-located. What our synthesis then adds is that contexting is inherently part of KT activities, and not something that should *also *be done. Insight into the activity of contexting can help identify what is necessary to create a network that supports and sustains the KT practices.

###  Institutionalising

 The health policy and systems research literature and the STS literature provide different accounts of the role of institutions in sustaining KT practices. Within the health policy and systems literature, institutions are often seen as relatively stable and durable structures that are necessarily social in nature.^8,62^ In contrast, the STS literature less explicitly focusses on institutions as subject of analysis. Instead, most of the STS literature builds on post-structuralist philosophy to describe how phenomena like infrastructures and networks can work institutionally. STS descriptions of institutions have in common that they conceptualise them as inherently unstable, dynamic, and mediated through materiality.^43,63-66^

 In the health policy and systems research literature, institutionalisation is seen as a way to make KT practices sustainable.^67^ Institutionalisation, in this case, equals a process where KT practices are linked to specific institutions, or the construction of new institutions (eg, regulation that requires health policies to be evidence-informed, see Ongolo-Zogo et al,^10^ and Tricco et al^6^). The idea is that this process of institutionalisation provides the KT practices with a certain “staying power.”^5,68^ Davies and Edwards,^5^ quoting Goodman and Steckler,^69^ use the notion of ‘staying power’ to connote the “endurance of change” and how that change “becomes part of everyday activities or normal practices in an organization.” How institutionalisation for KT practices can be achieved remains largely unclear, as most of the health policy and systems research literature is concerned with studying the extent to which KT practices have already been institutionalised and what institutional factors may have facilitated that process. There is little health policy and systems research into the relatively mundane work that is required to institutionalise KT practices.

####  Working With Institutions

 Through our review, a different understanding of institutions and institutionalism emerges. This sociological understanding builds on how Lascoumes and Le Gales^66^ write about institutions. In their descriptions, institutions are seen as a “more or less coordinated set of rules and procedures that governs the interactions and behaviours of actors and organisations.”^66^ The emphasis in this sociological understanding is on the fact that institutions may sometimes be less coordinated, and that they *govern* – instead of structure – behaviours of actors. This understanding thus moves away from seeing institutions as structures that act as facilitators or barriers of human behaviour only. Instead, following Colyvas and Jonsson^70^ institutions work as temporary fundaments that provide (new) practices with some stability. It is this dynamic approach to institutions that may help in understanding how KT practices can be sustained: the extent to which institutions aid the sustaining of KT practices is defined in how KT actors *work* with institutions. We will expand on this understanding in the paragraphs below.

 Most of the STS literature does not explicitly write about institutions, but focusses on how other phenomena can work institutionally. The idea of this shift is that focussing on what institutions precisely are is less productive than showing how some compositions can provide a (temporary) fundament to activities. A concept that is commonly used to describe such fundaments is that of infrastructures. With infrastructures, the STS literature refers to “the prior work (be it building, organization, agreement on standards, and so forth) that supports and enables the activity we are really engaged in doing.”^71^ These infrastructures are not backgrounded, but very much entangled with the practices that they provide a fundament to.^72,73^ More importantly, the focus is on how such infrastructures can be made and used.^73^ The implication of this perspective for the health policy and systems research literature is a shift from looking at institutionalisation (as outcome) of KT practices towards better understanding how the institutionalising (as activity) of KT practices works.

 There is a wide literature that specifically seeks to understand how actors work with institutions. This literature on ‘institutional work’ is not specifically part of STS literature, but institutional work is increasingly considered in empirical studies of STS-associated scholars (eg, Wallenburg et al^74^ and van de Bovenkamp et al^75^). Institutional work literature moves away from conceiving institutions as static compositions, and instead focusses on the work that actors put in creating, sustaining, and disrupting institutions.^76^ In this sense, institutions are strategically used to pursue objectives (cf. Callon^77^). Bijker et al^78^ for instance showed how the status of a National Health Council as prestigious advisory institute bestows legitimacy upon the advises it puts forth. Similarly, Van de Bovenkamp et al^75^ showed how hospital directors do institutional work on a daily basis, for instance by using the Healthcare Inspectorate to settle debates between medical specialists. In short, this literature on institutional work holds that the role of institutions, and how stable or durable they are, is defined in how actors interpret and work with institutions.^76,79,80^

####  Institutionalising and the Sustaining of Knowledge Translation Practices

 Following the STS perspective on institutions, we may conclude that institutionalising KT practices involves actively and strategically using institutions to sustain KT practices. The key difference between this perspective and how the health policy and systems research literature commonly writes about institutionalisation is the emphasis on how KT actors *work with* institutions in their daily practices. The emphasis is not on the process of institutionalisation at such, but on how KT actors use institutions such as academia, medicine, or advocacy groups to continuously be able to affect health policy or clinical practice. In short, the sustaining of KT practices depends partly on the extent to which KT actors use institutions to make and keep their KT practices productive.

## Discussion

 The aim of this CIS was to identify and explain those processes, activities, and efforts that facilitate the sustaining of KT practices in health policy-making processes. In our CIS, we reviewed the health policy and systems research literature and the STS literature that focussed on sustainability and KT. The main finding of our CIS is that common perspectives on the sustainability of KT practices focus on descriptions of end-states. Such descriptions offer important insight into what sustainability of KT practices looks like, but impair our understanding of how such states of sustainability are achieved and maintained. Thus, the synthesising argument of this CIS is that *conceptualisations of sustainability of KT practices would benefit from a shift from viewing sustainability as an end-state towards sustaining as the (often mundane) work that is required to make and keep KT practices productive*. In the literature we noticed that this *sustaining work* can be divided into three work processes. Our proposition is that these processes of *translating*, *contexting*, and *institutionalising* together can both explain and guide the sustaining of KT practices.

 The first sustaining work process that we described in our synthesis was that of translating. We showed how the literature describes that translating involves both transformation of knowledge and the creation of connections. Traditionally, the focus of studies on KT is mainly on how (scientific) knowledge is transformed as to make it utilisable. Here, most emphasis is placed on the ‘packaging’ of such knowledge, often referred to by terms such as ‘knowledge product’ or ‘tool.’^81^ Less emphasis is on the connections that are sought to carry such knowledge products and particularly the process in which such connections are made and maintained. Our synthesis suggests that this second element to translating is an important part of keeping KT practices productive. The observation that (social) connections between KT actors and the communities they work with are important is not new to the health policy and systems research literature,^82,83^ but the extent to which these connections affect the actual sustaining of KT practices remains undervalued.^84-86^

 Beside the process of translating, we also identified the process of contexting and institutionalising as important elements of sustaining work. Contexting of KT practices refers to the ongoing work of actors directed at* constructing contexts that work* and enrolling these contextual elements in such a way that their practices remain productive (cf. Borst et al^87^). This understanding moves away from context as a list of factors and instead proposes to disentangle how certain interventions or practices tie into their wider environment. In case of KT practices, this is often about creating ‘patchworks’^61^ of actors that can support the KT practices. An example could be how KT actors work to combine funding from different projects to sustain their core activities,^88^ or how policy-makers are engaged early on in a KT process as to create ownership and political buy-in.^10^ Finally, institutionalising KT practices refers to the strategic use of institutions as to create a (temporary) fundament on which KT practices can be organised. This institutional work offers the KT practices a certain durability by creating a relatively protected ‘environment’ that is less prone to political tides and financial instabilities.^89,90^ This environment itself can be actively constructed by situating KT practitioners within institutions such as academia or medical practice, or by creating productive dependencies with institutions that provide the KT practitioners with legitimacy.

###  Reflection on the Critical Interpretive Synthesis Approach

 Our review aimed to create a more dynamic and practice-centred theoretical perspective on the sustainability of KT practices by enriching the health policy and systems research literature with STS literature on sustainability and KT. We used a CIS approach to reviewing these literatures, because this approach is particularly useful for synthesising heterogenous literatures with disputes over certain concepts, and when the aim is to build new theory. Our particular use of the CIS approach presented several limitations. First, a large part of the STS literature is published in books and essays that are not necessarily indexed in scientific search engines. We attempted to overcome this issue by using expert suggestions and by reviewing all available handbooks on STS. Despite repeated invitations, not all contacted experts responded to our requests and some of their contributions were more extensive than others. Second, our use of handbooks to arrive at key STS insights may have steered the review away from more recent insights in this literature. However, both limitations were transcended as much as possible by triangulating data sources. Besides, as KT has been a central theme of the STS literature since the onset, it seems less likely that recent developments in STS would alter the overall contribution of that literature.

 In addition to forementioned reflections, our review approach also presented a more methodological challenge to attempting to synthesise insights from two disparate literatures. As with most bodies of literature, the boundaries are subject to how scholars work and reproduce them.^91^ In delineating STS and health policy and systems research, we ourselves created a binary which may not always be that clear-cut. There are several traditions within health policy and systems research where more socially scientific infused theories are used to understand, and practice, KT, these include integrated KT^86^ and complexity sciences.^92^ Scholars in these traditions translate, much like ourselves, concepts and frameworks between the different fields. Yet, we argue that the fields remain positioned on different sides of an ‘epistemic divide.’ STS scholars position themselves on the constructivist side of the divide and understand KT as a situated and contingent practice in which new connections between different actors are constantly (re)made – and that knowledge is shaped in this process. Scholars in health policy and systems research, including more social scientific extensions of that field, commonly use a realist epistemology and emphasise that KT is about using ‘rigorous’ methodology to objectively produce scientific knowledge that is equipped for informing (and improving) policies and independent of social relations. The latter understanding is thus much more restrictive and normative about what KT ‘is.’ Besides, STS scholars would argue that knowledge/policy interactions largely work through more relational, mundane, and unstructured practices.^93^ We see this epistemic divide as an important argument in favour of doing syntheses like the current one, where we may “foster conceptual and empirical cross-pollinations”^94^ between health policy and systems research and STS.

 A final potential limitation concerns the absence of a published protocol, or registration, prior to conducting this review. While we did construct a protocol and registered this protocol as part of our university regulations, it is common practice in health policy and systems research to publish such a protocol in a scientific journal or online registration service (eg, PROSPERO). A prominent logic behind this practice is the reduction of publication bias and increase of the study’s replicability. While this can be relevant for systematic reviews, we argue that this logic does not fit a review practice that is mostly interpretive and iterative and thereby inherently impossible to replicate in full.

###  Recommendations for Further Study

 Following the sustaining work perspective we developed in this CIS, and its emphasis on three core processes, we suggest three concrete recommendations for empirical research. The first recommendation concerns empirical study of how KT actors work with institutions. In contemporary health policy and systems research, much use is made of institutional theory to better grasp institutionalisation of, among other things, knowledge platforms.^62^ Our synthesis suggests a focus on how institutions are strategically used instead.^75,95^ This involves studying what actors do to create, change, or dispute institutions. The second recommendation for research concerns studying the way actors work with context in practice. In a similar way to institutional work, we think it can be valuable to see how and why actors designate things a role as context (cf. Kleinhout-Vliek et al^96^). We anticipate that situated descriptions of contexting may be translated into capacity-building workshops for KT actors. For instance, by educating KT actors on the importance of building relationships with key actors, or how to tinker with project funding. Finally, we envision using the notion of translating to map how KT actors construct networks and translate knowledge in practice, and to draw further lessons from these efforts (cf. Borst et al^87^).

## Conclusion

 The aim of this CIS was to identify and explain those processes, activities, and efforts that facilitate the sustaining of KT practices in health policy-making processes. The CIS has resulted in a new perspective on sustaining KT practices that shifts from sustainability as an end-state towards sustaining as the (often mundane) work that is required to make and keep KT practices productive. We have described this perspective as sustaining work to emphasise the practical efforts that are necessary to sustain KT practices. In the literature, we identified three processes of sustaining work: (*i*) translating, (*ii*) contexting, and (*iii*) institutionalising KT practices. Our suggestions are that these processes can guide empirical study of sustaining work and that these empirical insights, combined with this CIS, can inform training programmes for KT actors.

## Acknowledgements

 The authors would like to express their gratitude to Ruth Stewart, Andrea Wojcik, Maarten Kok, Martin Meremikwu, and Tom van der Grinten for their constructive comments on earlier versions of this manuscript.

## Ethical issues

 Not applicable.

## Competing interests

 Authors declare that they have no competing interests.

## Authors’ contributions

 RAJB wrote the first draft of the manuscript and revised the manuscript accordingly. All authors were involved in selecting and reviewing the manuscripts for the review, discussing the results, and contributed to the final manuscript.

## Funding

 This work was supported by the Netherlands Organisation for Scientific Research through its WOTRO Science for Using Research Evidence (SURe) programme (W 08.117.103).

## Supplementary files

Supplementary file 1. Search phrases and Eligibility Criteria.
