# Supplementary material for: Sustaining Knowledge Translation Practices: A Critical Interpretive Synthesis
Source: Int J Health Policy Manag. 2022 Feb 21;11(12):2793–804. doi: 10.34172/ijhpm.2022.6424 (PMC10105179; doi:10.34172/ijhpm.2022.6424)
Supplement: Supplementary file 1 — Search phrases and Eligibility Criteria. [file ijhpm-11-2793-s001.pdf]

Article title: Sustaining Knowledge Translation Practices: A Critical Interpretive Synthesis

Journal name: International Journal of Health Policy and Management (IJHPM)

Authors' information: Robert A.J. Borst*, Rik Wehrens, Roland Bal

Erasmus School of Health Policy & Management, Erasmus University Rotterdam, Rotterdam, The Netherlands.

(corresponding author: borst@eshpm.eur.nl)

Supplementary file 1: Search phrases and Eligibility Criteria

Cycle 1, step 1. Identifying existing systematic reviews

First, we search the PubMed database to identify existing systematic reviews. We activate the following filters:

- Article type: systematic reviews (as we are interested in reviews and not individual studies)
- Publication dates: published in the last 10 years, or 13 years when updated (as we are interested in the state-of-the-art)

First search:

- “knowledge translation” AND “health” AND “policy” (153 results) ("knowledge translation"[All Fields] AND "health"[All Fields] AND "policy"[All Fields] AND (systematic[sb] AND "2007/12/02"[PDat] : "2020/12/31"[PDat]))
- “knowledge translation” AND “health” AND “practice” (332 results) ("knowledge translation"[All Fields] AND "health"[All Fields]) AND "practice"[All Fields] AND (systematic[sb] AND "2007/12/02"[PDat] : "2020/12/31"[PDat])

Second search:

- ("knowledge translation"[Title] AND "review"[Title]) AND "health"[Title/Abstract] AND (systematic[sb] AND "2007/12/02"[PDat] : “2020/12/31”[PDat]) (41 results)
- ("research use"[Title] AND "review"[Title]) AND "health"[All Fields] AND (systematic[sb] AND "2007/12/02"[PDat] : “2020/12/31”[PDat]) (4 results)
- ("evidence use"[Title] AND "review"[Title]) AND "health"[All Fields] AND (systematic[sb] AND "2007/12/02"[PDat] : “2020/12/31”[PDat]) (3 results)
- ("research synthesis"[Title] AND "review"[Title]) AND "health"[All Fields] AND (systematic[sb] AND "2007/12/02"[PDat] : “2020/12/31”[PDat]) (2 results)
- (("research for policy"[Title]) AND "review"[Title]) AND "health"[All Fields] AND (systematic[sb] AND "2007/12/02"[PDat] : “2020/12/31”[PDat]) (0 results)
- (("knowledge for policy"[Title]) AND "review"[Title]) AND "health"[All Fields] AND (systematic[sb] AND "2007/12/02"[PDat] : “2020/12/31”[PDat]) (0 results)
- ("evidence for policy"[Title] AND "review"[Title]) AND "health"[All Fields] AND (systematic[sb] AND "2007/12/02"[PDat] : “2020/12/31”[PDat]) (1 result)
- ("research into action"[Title] AND "review"[Title]) AND "health"[All Fields] AND (systematic[sb] AND "2007/12/02"[PDat] : “2020/12/31”[PDat]) (0 results)

We will include papers if they meet the following criteria:

- The paper is based on a systematic review
- The paper addresses knowledge translation strategies and/or knowledge translation frameworks
- The paper focuses on healthcare as a general domain
- In general, papers will only be included if they meet all three criteria. Additional papers will be included, however, if they are particularly relevant based on some of these criteria, even if they do not meet all criteria. Examples are:
- A narrative review that does not qualify as systematic but does focus on knowledge translation frameworks in healthcare

- A systematic review of knowledge translation frameworks in which healthcare is one, but not the only domain.
- A narrative review that focuses on knowledge translation in relation to low- and middle-income countries

We have established the following exclusion criteria:

- Articles focusing on particular areas of clinical practice (as the aim of this first stage of the review is to identify key reviews on knowledge translation in health policy and practice in general as opposed to in particular clinical domains, such as primary care or mental health)
- Articles focusing on the best ways to conduct different kinds of reviews for health policy makers (as the focus is not on describing the best review methodology, but on identifying current state of knowledge on knowledge translation)
- Articles focusing on related concepts such as knowledge brokering or collaboration (except when explicitly linked to knowledge translation)
- Articles evaluating particular training models for practitioners or policy makers to increase knowledge translation
- Articles presenting particular approaches to provide evidence synthesis to policy makers or practitioners (as the aim of this search is to identify relevant systematic reviews on knowledge translation for policy and practice, not to identify singular approaches that have been developed)
- Articles addressing particular knowledge translation tools, such as ‘briefing notes’ or ‘toolkits’ (because the aim is to identify what is known about knowledge translation in general, not about specific techniques or instruments)
- Articles only presenting a protocol for a review (as these do not report on any results)

Cycle 1, step 2. Case-studies on KT in LMICs

First search via PubMed:

- "knowledge translation"[All Fields] AND "low- and middle income countries"[All Fields] AND (systematic[sb] AND "2007/12/02"[PDat] : "2020/12/31"[PDat]

To make sure we do not miss relevant studies, we will separately search for all articles containing a combination of the terms "knowledge translation" and the country names for all countries defined by the World bank in 2017 as low- and middle-income countries.1

Cycle 1, step 3. Inclusion of relevant conceptual articles

First search Web of Science and Google Scholar:

- "knowledge translation" AND "literature review" OR "definition" OR "defining" OR "conceptualizing" OR "concept" OR "meaning".

We use the following inclusion criteria:

- The papers should be highly influential (+ 200 citations in Google Scholar)
- The papers should make an explicit attempt to summarize/bring together/contrast different definitions or conceptual usages of knowledge translation or related terms from different disciplinary traditions
- The papers should be up to date, i.e. no more than 15 years old[2]

[1] These are: Afghanistan; Albania; Algeria; American Samoa; Angola; Argentina; Armenia; Azerbaijan; Bangladesh; Belarus; Belize; Benin; Bhutan; Bolivia; Bosnia and Herzegovina; Botswana; Brazil; Bulgaria; Burkina Faso; Burundi; Cabo Verde; Cambodia; Cameroon; Central African Republic; Chad; China; Colombia; Comoros; Congo, Dem. Rep.; Congo, Rep.; Costa Rica; Cote D'Ivoire; Cuba; Djibouti; Dominica; Dominican Republic; Ecuador; Egypt, Arab Rep.; El Salvador; Equatorial Guinea; Eritrea; Ethiopia; Fiji; Gabon; Gambia; Georgia; Ghana; Grenada; Guatemala; Guinea; Guinea-Bissau; Guyana; Haiti; Honduras; India; Indonesia; Iran, Islamic Rep.; Iraq; Jamaica; Jordan; Kazakhstan; Kenya; Kiribati; Korea, Dem. People's Rep.; Kosovo; Kyrgyz Republic; Lao PDR; Lebanon; Lesotho; Liberia; Libya; Macedonia, FYR; Madagascar; Malawi; Malaysia; Maldives; Mali; Marshall Islands; Mauritania; Mauritius; Mexico; Micronesia, Fed. STS.; Moldova; Mongolia; Montenegro; Morocco; Mozambique; Myanmar; Namibia; Nepal; Nicaragua; Niger; Nigeria; Pakistan; Panama; Papua New Guinea; Paraguay; Peru; Philippines; Romania; Russian Federation; Rwanda; Samoa; Sao Tome and Principe; Senegal; Serbia; Sierra Leone; Solomon Islands; Somalia; South Africa; South Sudan; Sri Lanka; St. Lucia; St. Vincent and the Grenadines; Sudan; Suriname; Swaziland; Syrian Arab Republic; Tajikistan; Tanzania; Thailand; Timor-Leste; Togo; Tonga; Tunisia; Turkey; Turkmenistan; Tuvalu; Uganda; Ukraine; Uzbekistan; Vanuatu; Venezuela, RB; Vietnam; West Bank and Gaza; Yemen, Rep; Zambia; Zimbabwe

[2] There is no specific conceptual argument underlying this cut-off point, but a 'rule of thumb' in which we try to balance practical workability (i.e. not looking back too far historically) with the aim to not miss out on some highly relevant articles we know from expertise in the field (such as the two examples articles). The cut-off point of 15 years seemed a good compromise.

Cycle 2. Map relevant literature in Science & Technology Studies

The second step consists of mapping relevant literature in the field of Science & Technology Studies (STS), with a particular focus on the question what (theoretical) insights have been developed about "knowledge translation" and what these insights can add to existing literature.

The results of cycle 1 will be a background document that will feed into this step. In particular, the results of cycle 1 will allow us to specify more precisely:

- what is currently known about knowledge translation (strategies) for policy and/or practice in the health domain
- to what extent do we know whether these knowledge translation strategies are applicable to low- and middle-income countries and to what extent different strategies are needed
- how knowledge translation is generally conceptualized in the health domain

This allows us to produce a specific problem analysis that serves as a point of comparison with insights derived from the field of STS. This is an interdisciplinary field that emerged during the 1970s and combines ideas from anthropology, history, philosophy, and sociology to study how society, politics, and culture affect scientific research and technological innovation, and how these, in turn, affect society, politics and culture. Gradually the field has expanded to include important sub-streams on innovation studies and policy sciences.

Selection of relevant chapters from handbooks:
These are the three handbooks that will be reviewed for relevant chapters:

- Jasanoff, S., Markle, G. E., Peterson, J. C., & Pinch, T. (Eds.). (1995). Handbook of science and technology studies. Sage publications.
- Hackett, E. J., Amsterdamska, O., Lynch, M., & Wajcman, J. (2008). The handbook of science and technology studies. The MIT Press.
- Felt, U., Fouche, R., Miller, C.A. & Smith-Doerr, L. (2016). The Handbook of Science and Technology Studies. The MIT Press.

Expert interviews:

The selection of experts is based on three criteria: 1) their prominence in the field; 2) the extent to which their work focuses on/is situated in low- and middle-income countries; 3) the extent to which the expert represents a perspective from low- and middle-income countries. For each criterion, we grade experts in three categories: 'V' refers to the criterion being somewhat met, 'VV' refers to the criterion being mostly met; 'VVV' refers to the criterion being fully met. We did not further specify the criteria into a further matrix, but more informally assessed them (for instance, for the criterion of 'prominence in the field', we judged the number of books, publications, and citations in the field, as well as the institutional position and the period of time working in the field, without quantifying these aspects as they serve as indicators rather than stringent criteria). As it is highly unlikely to find respondents that score excellent on all three criteria, we aim to find a reasonable balance of perspectives in our overall selection.

Name:	Prominence in the field	Work focused on LMIC:	Representing perspective from LMIC
Prof. Dr. Ir. Wiebe Bijker	VVV	VVV	
Prof. Harry Collins	VVV	V	
Prof. Arie Rip	VVV	VV	
Prof. Ulrike Felt	VVV	VV	
Prof. Dave Guston	VVV	VV	
Dr. Annapurna Mamidipudi	V	VVV	VVV
Dr. Teun Zuiderent-Jerak	VV	VV	
Prof. Sally Wyatt	VV	V	
Prof. Nelly Oudshoorn	VV	V	
Prof. Shiv Visvanathan	VV	VVV	VVV
